# Phenotypic Changes in T and NK Cells Induced by Sputnik V Vaccination

**DOI:** 10.3390/vaccines11061047

**Published:** 2023-05-31

**Authors:** Anna A. Boyko, Maria O. Ustiuzhanina, Julia D. Vavilova, Maria A. Streltsova, Sofya A. Kust, Andrei E. Siniavin, Irina V. Astrakhantseva, Marina S. Drutskaya, Elena I. Kovalenko

**Affiliations:** 1 Shemyakin & Ovchinnikov Institute of Bioorganic Chemistry, Russian Academy of Sciences, 117997 Moscow, Russialenkovalen@mail.ru (E.I.K.); 2 Center of Life Sciences, Skolkovo Institute of Science and Technology, 121205 Moscow, Russia; 3 N.F. Gamaleya National Research Center for Epidemiology and Microbiology, Ministry of Health of the Russian Federation, 123098 Moscow, Russia; 4 Division of Immunobiology and Biomedicine, Center of Genetics and Life Sciences, Sirius University of Science and Technology, 354340 Federal Territory Sirius, Russia; 5 Center for Precision Genome Editing and Genetic Technologies for Biomedicine, Engelhardt Institute of Molecular Biology, Russian Academy of Sciences, 119991 Moscow, Russia

**Keywords:** Sputnik V, T lymphocytes, NK cells, activating and inhibitory receptors, activation markers, proliferative senescence

## Abstract

A highly effective humoral immune response induced by the Sputnik V vaccine was demonstrated in independent studies, as well as in large-scale post-vaccination follow-up studies. However, the shifts in the cell-mediated immunity induced by Sputnik V vaccination are still under investigation. This study was aimed at estimating the impact of Sputnik V on activating and inhibitory receptors, activation and proliferative senescence markers in NK and T lymphocytes. The effects of Sputnik V were evaluated by the comparison of PBMC samples prior to vaccination, and then three days and three weeks following the second (boost) dose. The prime-boost format of Sputnik V vaccination induced a contraction in the T cell fraction of senescent CD57^+^ cells and a decrease in HLA-DR-expressing T cells. The proportion of NKG2A^+^ T cells was down-regulated after vaccination, whereas the PD-1 level was not affected significantly. A temporal increase in activation levels of NK cells and NKT-like cells was recorded, dependent on whether the individuals had COVID-19 prior to vaccination. A short-term elevation of the activating NKG2D and CD16 was observed in NK cells. Overall, the findings of the study are in favor of the Sputnik V vaccine not provoking a dramatic phenotypic rearrangement in T and NK cells, although it induces their slight temporal non-specific activation.

## 1. Introduction

The Sputnik V (Gam-COVID-Vac) vaccine in a heterogeneous two-dose (prime-boost) format was developed and first authorized in the Russian Federation [1]. It is based on the human recombinant replication-defective adenovirus vectors with serotype variants rAd26 and rAd5, injected sequentially with a three-week interval. Such approach allows to minimize adaptive immune response against adenovirus antigens. Along with other viral vector vaccines against COVID-19, Sputnik V effectively stimulates the production of specific antibodies: Sputnik V displayed a 91.6% efficacy against infection with Wuhan strain of SARS-CoV-2 and COVID-19-related death in the phase III clinical trial [2]. Later, its potency was confirmed in a large population involving more than 800 thousand vaccinated individuals in Hungary [3], and comparable results were obtained after the boost dose administration during the dominance of the SARS-CoV-2 Delta variant [4]. This vaccine was distributed in more than 60 countries, and its effectiveness has been proved among different cohorts in several countries [5,6,7]. The virus-neutralizing activity of RBD-specific IgGs induced by the Sputnik V vaccine was shown against local and worldwide SARS-CoV-2 variants of concern (VOC) [8,9] and had a comparable rate of neutralization with mRNA vaccines [10].

Cellular immunity changes associated with anti-SARS-CoV-2 vaccines Moderna mRNA-1273, Pfizer BioNTech (BNT162b2), Janssen Ad26.COV 2.S and ChAdOx1 nCoV-19 Oxford were extensively explored [11,12,13,14,15,16], while less is known about cell-mediated immunological shifts driven by the Sputnik V vaccination. It is known that, along with other viral vector vaccines, Sputnik V is able to induce a cell-mediated immune response and elevate IFN-γ production in PBMC, affecting CD8^+^ and CD4^+^ T cells [17,18,19].

The long-term potency of the Sputnik V vaccine in activating T cell immune response was reported earlier [20]. Sustained Sputnik-V-induced activation of B cell immunity and accumulation of memory B cells in a cohort of vaccinated subjects has also been demonstrated, being heavily dependent on preexisting SARS-CoV-2 infection [21,22].

A robust response of the previously infected individuals to vaccination by mRNA vaccine suggests that naturally acquired immunity after COVID-19 may be sufficiently enhanced by vaccination [23]. SARS-CoV-2 infection prior to vaccination significantly increased antibody titers and neutralizing capacity following Sputnik V vaccination [5].

Along with high B-cell-mediated humoral responses and T-cell-mediated IFN-γ production after Sputnik V administration, the cytokine profile among vaccinated individuals was shown to be altered. A similar alteration in COVID-19 convalescents may indicate common mechanisms of the immune response activation against an intact virus and Sputnik V vaccine [20].

At the same time, it remains unclear whether Sputnik V vaccination promotes phenotypic changes in the T cell and NK cell subsets. The main goal of the current study was to determine alterations in the expression of activating and inhibitory receptors, markers of proliferative senescence and activation in NK and T cells after Sputnik V vaccination depending on the COVID-19 infection history.

## 2. Materials and Methods

### 2.1. Donor Characteristics

**Figure 1:**
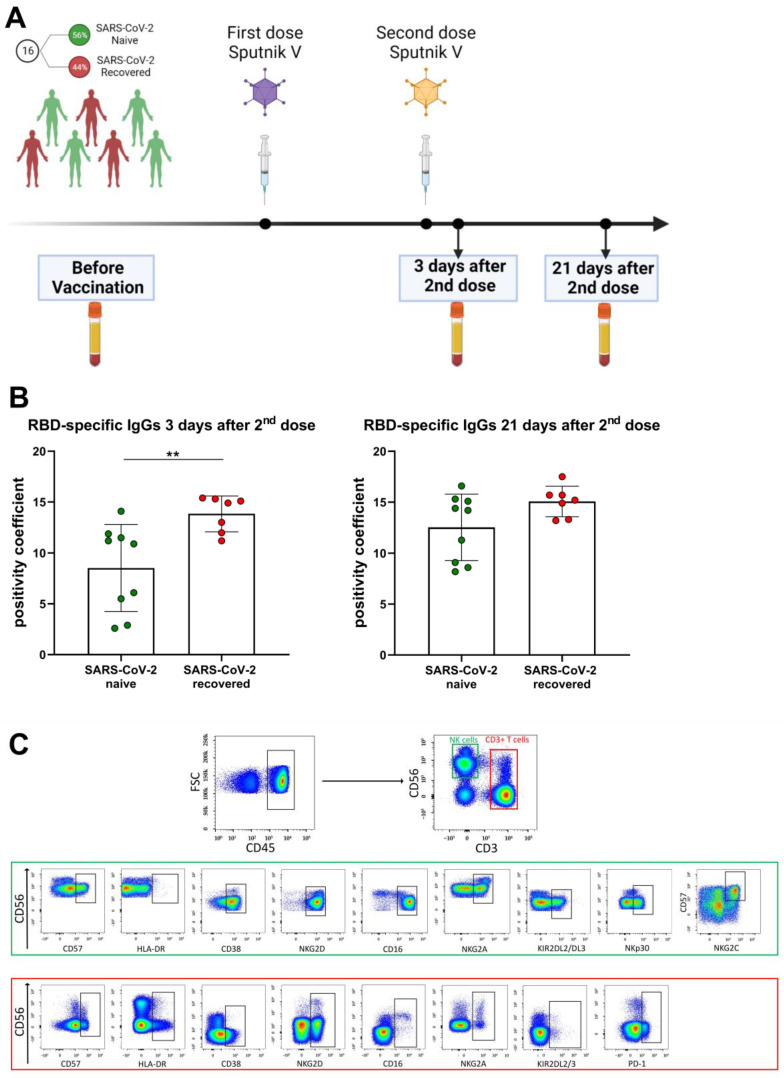
Study design, characteristics of SARS-CoV-2-specific antibody response of the volunteers and gating strategy for cytometric analysis. (**A**) Study design. (**B**) Levels of RBD-specific IgGs following the Sputnik V vaccination measure in 3 days and 21 days after the second dose administration. The positivity coefficient was calculated as a fold-increase relative to the negative cut-off value of the optical density in each ELISA measurement. Data are presented as mean ± SD and individual values; ** *p* < 0.01. (**C**) Surface expression of CD57, HLA-DR, CD38, NKG2D, CD16, NKG2A, KIR2DL2/DL3PD-1, PD-1, NKG2C, NKp30 in NK cells and/or T lymphocytes were analyzed by flow cytometry after staining with fluorescent-labeled specific monoclonal antibodies. NK and T cells were defined as CD3^−^CD56^+^ and CD3^+^, respectively, in CD45^high^ region. Representative staining data are presented.

Sixteen healthy individuals (aged from 28 to 73 years; median age—40 years; 6 male and 10 female) receiving first and second (boost) doses of the Sputnik V vaccine were independently recruited during April–September of 2021. All participants’ blood sera were analyzed for the presence of N-protein before the vaccination. Receptor-binding domain (RBD) specific IgGs against SARS-CoV-2 were measured before vaccination and 3 days and 21 days after the second dose (Figure 1A). Seven volunteers had recovered after mild COVID-19 symptoms (fever ≤ 39 °C, without pneumonia) no later than 4 months prior to vaccination and had positive levels of N-proteins and/or RBD-specific IgGs prior to vaccination (SARS-CoV-2-recovered group). Nine participants were naïve to SARS-CoV-2 infection since the SARS-CoV-2 N-protein and RBD-specific IgGs were not detected in their sera prior to the vaccination (SARS-CoV-2 naive group). The division of volunteers into the SARS-CoV-2-naive and SARS-CoV-2-recovered groups has been used in some analyses. Within 3 weeks after two doses of vaccination, no COVID-19 infection was recorded in any of the donors. The criteria for exclusion of volunteers from the study were symptoms of a cold and malaise before blood sampling.

### 2.2. Obtaining of Samples

To isolate the peripheral blood mononuclear cells (PBMC), blood samples were collected in EDTA-containing test tubes and centrifuged in a Ficoll gradient with 1.077 g/cm^3^ density (PanEco, Moscow, Russia). Serum samples were collected in tubes with gel-clot activators and stored at −60 °C until used. Obtained PBMCs were immediately analyzed by flow cytometry.

### 2.3. Analysis of Antibody Responses

RBD-specific IgG levels in healthy volunteers before vaccination, 3 days and 21 days after the second dose of Sputnik V vaccine were measured using ELISA (Vector-Best, Novosibirsk, Russian Federaion). Additionally, SARS-CoV-2-specific antibody response was assessed using MILLIPLEX^®^ SARS-CoV-2 Antigen Panel 1 IgG kit (cat.# HC19SERG1-85K, EDM Millipore, Germany) according to manufacturer’s instructions. Median fluorescence intensity (MFI) was registered using xPotent software (EDM Millipore, Germany, https://www.luminexcorp.com/eu/xponent/). The cut-off level was calculated for negative control serum according to the formula: Antigen MFI mean ± 3 SD. The data were analyzed as a signal to cut off ratio by GraphPad software (https://www.graphpad.com/), where a ratio greater than 1 was considered positive.

### 2.4. Phenotype Analysis

PBMC samples were stained with the following antibodies: CD3-PerCP clone HIT3a, CD3-APC clone SK7, CD45-PerCP clone 2D1, NKG2D-PE clone 1D11, KIR2DL2/DL3-FITC clone DX27, NKp30-PE clone P30-15 (all Sony Biotechnology, San Jose, CA, USA); CD3-APC-Vio770 clone REA613, CD56-Vio-Blue clone REA196, CD45-Vio-Green clone 5B1, CD14-PE-Vio-770 TÜK4, HLA-DR-PE-Vio-770 clon AC122, CD57-APC-Vio-770 clon TB03, NKG2C-FITC clone REA205, NKG2A-PE/PE-Vio615 clone REA110, CD16-APC clone REA423 (all Miltenyi Biotec, Bergisch Gladbach, Germany); CD38 (Beckman Coulter, USA); PD-1-Alexa Fluor 647 (clone EH12.2H7, BioLegend, San Diego, CA, USA). Samples were analyzed using a MACSQuant 10 flow cytometer (Miltenyi Biotec, Bergisch Gladbach, Germany) equipped with lasers λ = 405 nm, λ = 488 nm, λ = 635 nm; threshold was set to cut off events with low CD45 staining. The applied panels of fluorochrome-conjugated monoclonal antibodies to the listed immune cell surface markers are shown in Table 1. Samples were analyzed using a MACSQuant 10 flow cytometer (Miltenyi Biotec, Bergisch Gladbach, Germany) equipped with lasers λ = 405 nm, λ = 488 nm, and λ = 635 nm.

### 2.5. Statistical Analysis

The data were analyzed using FlowJo X 10.0.7r2 (FlowJo LLC, Ashland, OR, USA) and GraphPad Prism 8.00 ver. software (StatSoft Inc., Tulsa, OK, USA). Results are shown as the mean ± standard deviation unless otherwise indicated. Mann–Whitney U test was carried out for two independent cohorts. Nonparametric tests for all data were applied: Freedman signed rank test for multiple paired comparisons per family, and Mann–Whitney test for non-paired two-sample comparisons. The value of *p* < 0.05 was considered statistically significant. Data are presented as the mean ± SD, * *p* < 0.05, ** *p* < 0.01.

## 3. Results

### 3.1. Sputnik V Prime-Boost Vaccination Induces a Contraction of CD57^+^ T Cell Subset

All study participants were examined for SARS-CoV-2-specific IgGs prior to vaccination with Sputnik V. Based on the negative and positive pre-vaccination levels of the serum N-specific and RBD-specific SARS-CoV-2 IgGs, the cohort was divided into two groups: SARS-CoV-2-naive and SARS-CoV-2-recovered (Table 2). Specific T cell responses intensify greatly at day 28 ± 2 after the first Sputnik V dose injection [18] and this period falls on the first days after the second dose. To assess the effectiveness of vaccination, we measured the RBD-specific IgGs at two time points: 3 days and 21 days after the second (boost) dose of Sputnik V (Figure 1A). As expected, among the SARS-CoV-2-recovered group the level of RBD-specific antibodies 3 days after the Sputnik V second dose was higher than in the SARS-CoV-2-naive group, whereas 21 days after the boost dose the differences between the groups became non-significant (Figure 1B).

In this work, we focused on the analysis of phenotypic changes in NK and T cells in response to Sputnik V vaccination, regarding their degree of differentiation, the state of activation, and the levels of activating and inhibitory receptors. The gating strategy for determination of T lymphocytes, NK cells and surface marker levels is presented in Figure 1C. Prior to vaccination and in the designated time points after the second dose, the percentages of the T lymphocytes (CD3^+^), including CD56^−^ T cells and more differentiated CD56^+^ T cells (NKT-like cells), remained constant (Supplementary Figure S1A). Similar to T cells, the proportion of NK cells including less differentiated CD56^bright^ and more differentiated CD56^dim^ did not change (Supplementary Figure S1B).

**Figure 2:**
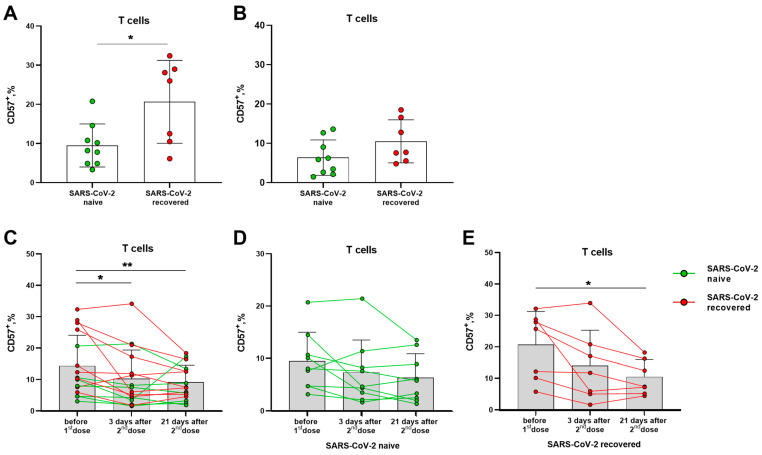
Alterations in terminally differentiated circulating T lymphocytes proportion induced by Sputnik V vaccination. (**A**) The frequencies (%) of CD57^+^ T cells before the vaccination in the SARS-CoV-2-naive and recovered donor groups (here and after, green and red, respectively). (**B**) Percentages of CD57^+^ T cells measured in 21 days after the second dose of Sputnik V. (**C**) The percentages of CD57^+^ T cells in 3 and 21 days after the second Sputnik V dose measured in all donors. (**D**,**E**) The percentages of CD57^+^ T cells in 3 and 21 days after the second Sputnik V dose measured separately in the SARS-CoV-2-naive and recovered groups, respectively. Data are presented as mean ± SD with individual values, values of the same donor are linked with color lines. * *p* < 0.05, ** *p* < 0.01.

Prior to vaccination, donors in the SARS-CoV-2-recovered group had a higher proportion of circulating terminally differentiated CD57^+^ T lymphocytes compared to naive volunteers (Figure 2A). In 21 days after the second vaccine dose, the levels of CD57^+^ T lymphocytes did not differ in the two compared groups. The differentiation profile of T lymphocytes was characterized by a gradual decrease in the proportion of CD57^+^ cells by the 21 day after the boost dose (Figure 2B,C), and the decrease remained significant in a smaller group of the SARS-CoV-2-recovered donors (Figure 2C,E), while in the naive group, the differences were not preserved (Figure 2D). Among NK cell subsets, the vaccination did not induce an alteration in the proportion of cells with the signs of terminal differentiation, and SARS-CoV-2 infection status did not affect the CD57 expression profile (Supplementary Figure S2).

Thus, Sputnik V vaccination was associated with a decrease in the proportion of circulating terminally differentiated T lymphocytes, most pronounced in the group of SARS-CoV-2-recovered donors, while no such decrease was recorded among NK cells.

### 3.2. Activation Profile of T and NK Lymphocytes after the Sputnik V Vaccine Administration

**Figure 3:**
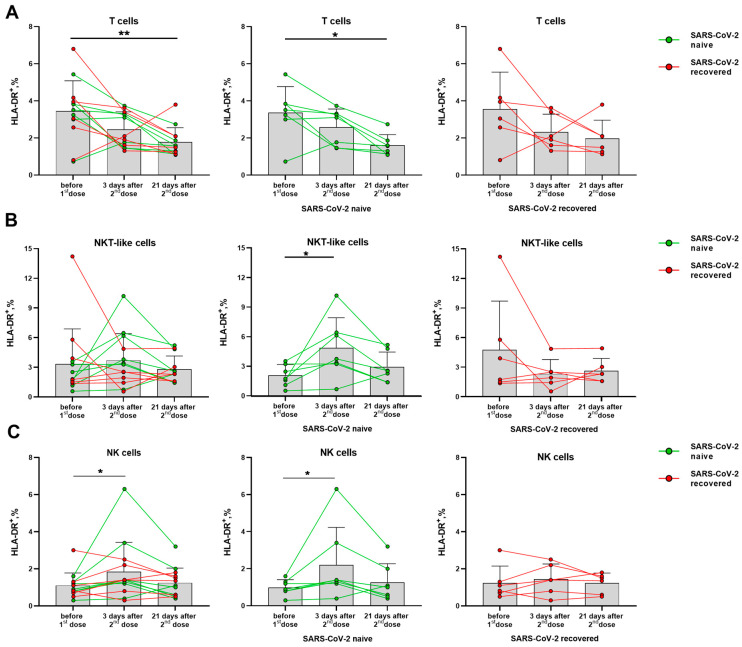
Changes in the HLA-DR surface expression on T lymphocytes and NK cells in 3 and 21 days after administration of the second dose of Sputnik V vaccine. (**A**) Proportions of HLA-DR^+^ T cells in all donor cohorts, and in the SARS-CoV-2-naive and recovered subgroups. (**B**) Proportions of HLA-DR^+^ cells in the NKT-like cell subset in all donors, and in the SARS-CoV-2-naive and recovered subgroups. (**C**) Proportions of HLA-DR^+^ cells in the NK in all donors, and in the SARS-CoV-2-naive and recovered subgroups. Data are presented as mean ± SD with individual values. Values from the same donor are linked by color lines. * *p* < 0.05, ** *p* < 0.01.

Activation degree of T cells and NK cells was assessed by the HLA-DR and CD38 markers. Among T lymphocytes, the vaccination was shown to be accompanied by a decreased HLA-DR expression level. The similar trends were observed in the groups of naive and recovered donors analyzed separately (Figure 3A). In the NKT-like cell subset, on the contrary, the HLA-DR dynamics was different for the SARS-CoV-2-naïve and SARS-CoV-2-recovered donors. In the naive donor group, the proportion of HLA-DR+ cells increased on day 3 after the second dose, and did not change significantly until day 21, while no increase in the HLA-DR+ NKT-like cell proportion was detected for the recovered donors (Figure 3B). The increase in the proportion of HLA-DR-expressing cells was also recorded for NK cells on day 3 after the second vaccine dose among the naive but not recovered donors (Figure 3C). Corresponding trends were observed in both CD56bright and CD56dim NK cell subsets (Supplementary Figure S3).

Thus, the SARS-CoV-2-naïve group differed from the group of recovered patients by the accumulation of HLA-DR+ cells among both NK and NKT-like cell subsets, together with a decrease in the level of HLA-DR-expressing cells among all T cells.

**Figure 4:**
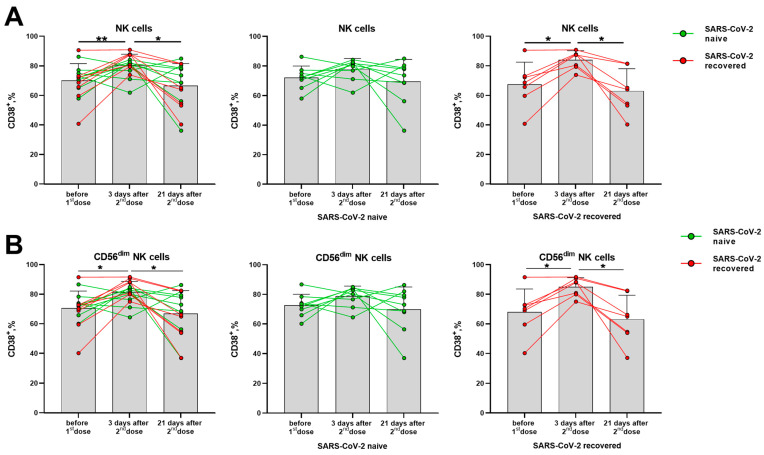
Changes in CD38 surface expression in NK cells in 3 and 21 days after the second dose of the Sputnik V vaccine in all donors, in the SARS-CoV-2-naive and recovered subgroups. (**A**) The proportion of CD38^+^ cells among the total NK cell pool. (**B**) The proportion of CD38^+^ cells in the CD56^dim^ NK cell subset. Data are presented as mean ± SD with individual values. Values from the same donor are linked by color lines. * *p* < 0.05, ** *p* < 0.01.

The CD38 molecule is robustly induced in immune cells during the activation, but its distribution in the lymphocytes differed from the HLA-DR molecule. In T cells (CD56^−^ and CD56^+^ NKT-like), CD38 expression levels have not changed significantly in response to the vaccination (Supplementary Figure S4A–C). In NK cells, a temporal increase in the proportion of CD38^+^ cells was observed in day 3 after the second vaccine dose, mostly due to alterations in the more differentiated CD56^dim^ cells (Figure 4A,B). In contrast to the proportion of the HLA-DR-expressing NK cells, which increased in the group of naive donors, augmentation of the CD38-expressing NK cell pool was more pronounced in the SARS-CoV-2-recovered group, both in total NK cell population and in the CD56^dim^ subset (Figure 4B,C). In less mature CD56^bright^ NK cells, no differences in CD38 expression were observed following vaccination (Supplementary Figure S4D).

Similar to HLA-DR, the CD38 expression increase seemed to reflect a short-term response to the vaccine, as the increased proportions of cells expressing these markers were observed 3 days after the boost dose, and then the values returned to the pre-vaccination levels (Figure 4A,B).

**Figure 5:**
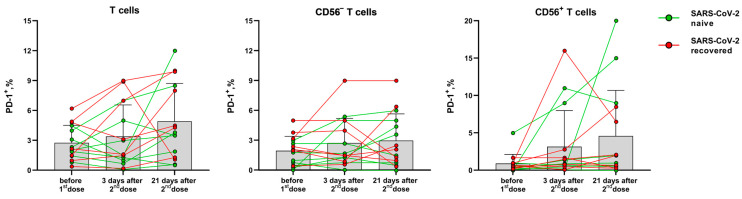
The proportion of PD-1-expressing T cells, CD56^−^ T cells and NKT-like cells before the vaccination and 3 and 21 days after the second dose of the Sputnik-V vaccine. Data are presented as mean ± SD with individual values. Values from the same donor are linked by color lines.

In contrast to the activation markers HLA-DR and CD38, PD-1 expression on T cells is associated with their exhaustion during immune response [24]. The proportion of T cells (CD56^−^ and NKT-like) expressing the PD-1 inhibitory receptor did not change significantly after vaccination compared to the pre-vaccination level. The PD-1 expression level dynamics varied greatly between the donors, and an increase in PD-1 level was observed in T cells of some donors 21 days after the second dose (Figure 5).

Thus, in contrast to T lymphocytes, the phenotype of NK cells indicates their activation on the 3rd day after the administration of the second vaccine dose: via an increase in HLA-DR^+^ cells in the group of SARS-CoV-2-naive donors, and via an increase in CD38 expression level in the group of SARS-CoV-2-recovered donors. The activation of NK cells was not accompanied by a vaccine-mediated induction of their differentiation. NKT-like cells showed HLA-DR expression level dynamics similar to those of NK cells.

### 3.3. Changes in the Activating and Inhibitory Receptor Expression on T and NK Cells after Prime-Boost Doses of the Sputnik V Vaccine

**Figure 6:**
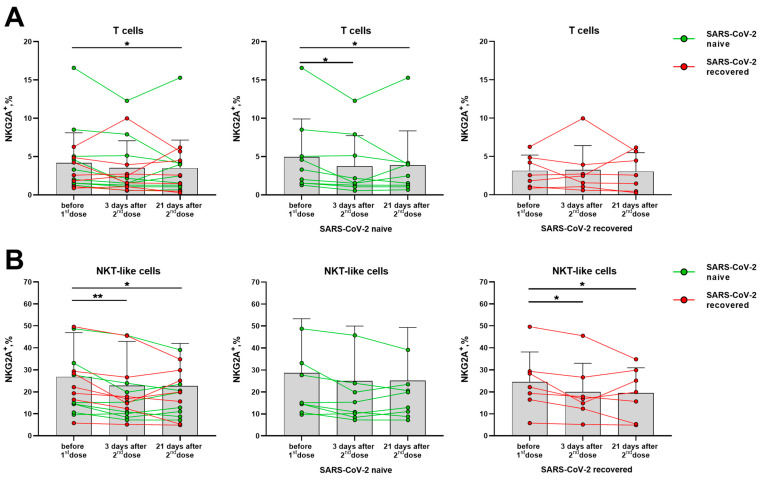
Changes in the surface expression of NKG2A on T cells and NKT-like cells in 3 and 21 days after the administration of the second dose of the Sputnik V vaccine in all donors, and in the SARS-CoV-2-naive and recovered subgroups. (**A**) The proportion (%) of NKG2A^+^ cells in the T cell subset. (**B**) The proportion (%) of NKG2A^+^ cells in the NKT-like cell subset. Data are presented as mean ± SD with individual values. Values from the same donor are linked by color lines. * *p* < 0.05, ** *p* < 0.01.

Although NK cells showed signs of activation after vaccination, the expression of the inhibitory receptors NKG2A and KIR2DL2/DL3 among NK cells and their subsets did not vary in the indicated post-vaccination period (Supplementary Figure S5A,B). In T cells, a decrease in NKG2A^+^ cell fraction was observed in samples from SARS-CoV-2-naive donors, while no changes were recorded in the SARS-CoV-2-recovered group (Figure 6A). However, among the NKT-like cell subset, a decrease in the proportion of NKG2A-expressing cells was revealed in the SARS-CoV-2-recovered group (Figure 6B).

**Figure 7:**
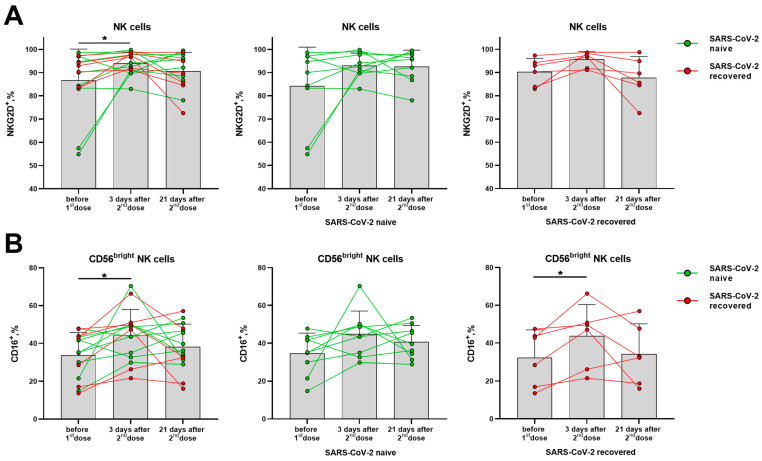
Changes in surface expression of NKG2D and CD16 on NK cells in 3 and 21 days after administration of the second dose of the Sputnik V vaccine in all the donors, and in the SARS-CoV-2-naive and recovered subgroups. (**A**) The proportion (%) of NKG2D^+^ cells in the NK cell subset. (**B**) The proportion (%) of CD16^+^ cells in the CD56^bright^ NK cell subset. Data are presented as mean ± SD with individual values. Values from the same donor are linked by color lines. * *p* < 0.05.

The proportion of NKG2D-expressing NK cells was significantly increased in the total cohort on day 3 after the second dose, although no significant differences were observed among donors with different SARS-CoV-2 backgrounds observed separately (Figure 7A). An increase in the proportion of CD16-positive NK cells was found among the less differentiated CD56^bright^ NK cells on day 3 after the second dose, which then declined by day 21 in some donors (Figure 7B). At the same time, in the populations of CD56^−^ T cells and NKT-like cells, the proportions of NKG2D^+^ and CD16^+^ cells remained the same (Supplementary Figure S6A,B).

Since NK cells responded to the administration of the boost dose of the Sputnik V vaccine by increasing the NKG2D- and CD16-activating receptors expression, the post-vaccination NK cell response was also analyzed for the expression of the NKp30 and NKG2C receptors—the most specific for this cell type.

Analysis of CD337 (NKp30) expression, a trigger molecule involved in the natural cytotoxicity of NK cells, did not show an increase in the proportion of NKp30^+^ cells among either CD56^bright^ or CD56^dim^ subsets in the analyzed time intervals following boost vaccination (Supplementary Figure S7A). This receptor is expressed to a different extent not only during cell proliferation, but also in resting NK cells. However, we did not detect any upregulation of the NKp30 receptor expression per cell estimated by median fluorescence intensity (MFI) normalized to auto-fluorescence control (Supplementary Figure S7B).

Similar to NKp30, the level of NKG2C-expressing NK cells has not significantly changed after vaccination in the total cohort (Supplementary Figure S8A). The proportion of NKG2C^+^CD57^+^ NK cells with adaptive or memory-like properties remained constant for 3 weeks after the second dose of the vaccine (Supplementary Figure S8B).

Thus, the expression of the inhibitory receptors NKG2A and KIR2DL2/DL3 on NK cells was not altered by vaccination, while the fractions of cells, which expressed the activating receptors NKG2D and CD16, on the contrary, were slightly increased. Among T cells of the SARS-CoV-2-naive donors, a decrease in the proportion of NKG2A^+^ cells was observed. In NKT-like cells, on the contrary, the NKG2A^+^ cell fraction decrease was recorded in the SARS-CoV-2-recovered donor group.

Taken together, our results suggest that the Sputnik V vaccination slightly affects NK and NKT-like cells in terms of their activation in the absence of shifts in the differentiation, while in T cells, the vaccination induces an increase in the proportion of replicative active CD57^−^ cells, which is more pronounced in individuals that recovered from SARS-CoV-2 infection.

## 4. Discussion

An extent to which Sputnik V vaccination can cause phenotypic changes among T cells and NK cells, the main participants in cell-mediated antiviral immune response, is poorly understood. In this work, the expression of key receptors and signature molecules was studied among the three cell populations: conventional CD3^+^CD56^−^ T lymphocytes, CD3^−^CD56^+^ NK cells, and more differentiated T lymphocytes CD3^+^CD56^+^ (NKT-like cells), which share some features of both T cells and NK cells [25]. Vaccination with more than one dose is necessary for the formation of a more effective immune response to SARS-CoV-2. The Sputnik V vaccine was shown to induce a strong cellular immune response, characterized by the secretion of IFN-γ by PBMC upon the SARS-CoV-2 glycoprotein S stimulation in 28 ± 2 days after the first vaccine dose, i.e., during first week after the second Sputnik V dose [18]. In this post-vaccination period, we could expect significant phenotypic alterations in either NK cells or T cells due to their activation.

CD57 expression in T cells and NK cells defines the terminally differentiated lymphocytes undergoing replicative senescence, most often due to repeated antigen stimulation (chronic stimulation). An acquisition of CD57 by these cells is associated with their higher effector functions, i.e., cytotoxic activity. Moreover, the part of CD57^+^ NK cells exhibit memory-like features [26]. Therefore, a change in the proportion of CD57^+^ cells in response to SARS-CoV-2 vaccination might be expected.

In our work, we did not find any transition of NK cells from the CD56^bright^ to CD56^dim^ phenotype after the Sputnik V vaccine administration, either in the group of naive or SARS-CoV-2-recovered donors. There were also no signs of the NK cell maturation from the CD56^dim^CD57^−^ to CD56^dim^CD57^+^ stage. At the same time, we observed a temporary activation of NK cells after the Sputnik V vaccination, via upregulation of HLA-DR and CD38 expression. Moreover, an increase in HLA-DR-expressing NK cells was found in the SARS-CoV-2-naive donor group, while the proportion of CD38-positive cells increased in the donors who have experienced SARS-CoV-2 infection. CD38 is a multifunctional adhesion molecule possessing ecto-enzymatic activity. In contrast to HLA-DR, CD38 is expressed on NK cells in a constitutive manner, but is upregulated upon the induction of NK cell effector response [27].

It was previously shown that NK cells can participate in the response to vaccination. The introduction of attenuated viral Ebola and yellow fever vaccines induced activation and differentiation of these cells [28,29,30]. Only a few studies have evaluated the effects of COVID-19 vaccines on NK cell phenotype and functions. It has been demonstrated that mRNA vaccine BNT162b2 can affect NK cells [31,32]. In particular, an absence of humoral response to the vaccine in patients with oncohematologic diseases was associated with a low NK cell proportion in their peripheral blood [33]. On the one hand, the BNT162b2 vaccine did not result in significant changes in NK cell function or phenotype (from pre-vaccination to post-vaccination) [16]. On the other hand, a transient activation of NK cells in first few days after the vaccination observed in our study was previously reported in several other works [32,34,35].

SARS-CoV-2 spike proteins were shown not to alter NK cell activation or modify NK cell cytotoxicity against K562 in vitro. On the other hand (in another model), SARS-CoV-2 peptides have shown an ability to bind to the NKG2D receptor, capable of directly targeting virus-infected cells, thus increasing NK cell activity [36]. On day 3 after the second Sputnik V dose, we recorded an increase in the proportion of NKG2D expressing cells, but it cannot be excluded that it was due to the adenoviral component of the Sputnik V vaccine.

In the antiviral response mediated by NK cells, a special role is given to the subset with the adaptive-like phenotype NKG2C^+^ and memory-like phenotype CD57^+^NKG2C^+^. There are a number of studies focusing on these NK cell types in COVID-19 and their response to the stimulation with the SARS-CoV-2 spike protein [36,37,38]. For example, usage of the BNT162b2 mRNA vaccine led to a peripheral blood pattern, in which an increased frequency of NKG2C^+^ NK cells correlated with high antibody titers in the post-vaccination period [16]. It is possible that the expansion of this subset could increase in response to the spike protein in a vaccine in the SARS-CoV-2-recovered donors. In our cohort, as expected, antibody responses to the Sputnik V vaccine were developing more slowly in naïve individuals than in previously SARS-CoV-2 infected volunteers, but the SARS-CoV-2 exposure status was not associated with a change in the proportion of NKG2C^+^ or CD57^+^NKG2C^+^ NK cells with memory-like properties (Supplementary Figure S7A,B).

One of the triggers for NK cell activation is a decrease in signals from inhibitory receptors, mainly NKG2A and KIRs. Both NKG2C and NKG2A recognize the HLA-E molecule. It was suggested that the SARS-CoV-2 spike protein 1, including the RBD domain, can control NK cell activation via HLA-E upregulation and modulation of NKG2A-expressing NK cells [39]. However, in the current work, both the proportions of NKG2A^+^ and KIR2DL2/3^+^ NK cells remained constant after the Sputnik V vaccination at the observed time points regardless of the naive or recovered status of the individuals.

NK cell subpopulations CD56^bright^ and CD56^dim^ differ in the degree of their differentiation and in the functional activity. CD56^dim^ NK cells demonstrate higher cytotoxicity and can perform antibody-dependent cell-mediated cytotoxicity (ADCC) due to the almost 100% expression of CD16 (FcγRIIIA receptor). In the CD56^bright^ subset, the expression of CD16 is low and does not exceed 30–50% of cells, these NK cells are less cytotoxic but respond to the cytokine stimulation by more intensive cytokine production [40]. CD16 expression changes during early NK cell activation and plays a role in the post-vaccination immunity. For example, two-dose heterologous Ebola virus vaccine regimen, adenovirus type 26. ZEBOV, stimulated a decrease in CD16 expression, possibly helping to reduce the response of NK cells and to maintain the immune potential of T lymphocytes [41]. The BNT162b2 SARS-CoV-2 mRNA vaccine was shown to induce ADCC reactivity mediated by CD56^dim^ NK cells [42]. In this study, we have recorded an increase in the proportion of CD16^+^ NK cells only in the CD56^bright^ subset in the group of SARS-CoV-2-recovered donors. In the absence of signs of NK cell differentiation from the CD56^bright^ to CD56^dim^ stage, the increase in CD16^+^CD56^bright^ NK cells can be interpreted as a shift from less mature CD56^bright^CD16^−^ cells to more mature CD56^bright^CD16^+^ cells able to exert the Fc-mediated effector functions.

Although we did not find any changes in the proportion of the most differentiated CD57^+^ NK cells following vaccination, a decrease in the proportion of CD57^+^ T lymphocytes was observed. Such a trend was also significant in a smaller cohort of the SARS-CoV-2-recovered patients analyzed separately. This can be caused by concomitant expansion of the pool of immature and, thus, more proliferative active CD57^−^ T cells, due to the T cell response to the vaccine administration in the previously recovered patients. It has been previously shown that mRNA vaccines induce an increase in the fraction of Spike-specific long-lived CD4^+^ T cells lacking CD57 in vaccinated SARS-CoV-2-recovered individuals compared to the previously uninfected individuals [43]. The second vaccine dose in the prime-boost format BNT162b2 vaccination resulted in a decrease in the most differentiated TEMRA cell proportion within a fraction of CD4^+^ T cells expressing activation-induced markers (AIM+) on day 14 after the vaccination [12]. Thus, a decrease in CD57^+^ T lymphocytes, which are often consistent with the TEMRA phenotype, indicates the increase in actively proliferating (CD57^−^) T cells, which happens in response to S-protein antigenic stimulation in different SARS-CoV-2 platforms.

In contrast to NK cells, conventional (CD56^−^) T lymphocytes did not show any signs of activation as assessed by elevating CD38 or HLA-DR expression following the second vaccine dose. The similar trends were observed in the groups of naive and recovered donors analyzed separately. HLA-DR can be expressed in both CD8^+^ and in CD4^+^ lymphocytes [44]. The role of HLA-DR on CD4^+^ T cells for antigen-presentation was evaluated [45]. The results strongly suggest that the CD4^+^ HLA-DR^+^ T cells possess properties of Tregs rather than T effector cells in antigen-specific immune response in vitro. Thus, the gradual decline of HLA-DR expression level among T lymphocytes on the 21st day after the second dose Sputnik V administration could be associated with down-regulation of the regulatory properties of T cells. After the BNT162b2 boosting, the transient increase in HLA-DR and CD38 expression levels was observed only in a part of T cells [12]

At the same time, we observed a decrease in the NKG2A^+^ T cell proportion in the group of SARS-CoV-2-naive donors. It is possible that such a decrease is associated precisely with an increased level of T cell activation though the mechanisms via HLA-E binding. However, the reasons and mechanisms underlying this phenomenon still need to be investigated. Thus, vaccination with Sputnik V resulted in a progressive contraction of the NKG2A^+^ T cell pool, which manifested itself in the group of naive donors. After virus antigen recognition and activation, T cells often up-regulate the expression of PD-1 to prevent an excessive response [46]. An increase in the expression of this receptor is associated with signs of immunosuppression and exhaustion and is recorded in severe cases of SARS-CoV-2 infection. Upregulation of the PD-1 expression following Sputnik V vaccination was observed only in some donors both among naive and recovered groups, suggesting that the degree of immune system activation and concomitant immune exhaustion induced by the vaccine are very individually regulated.

The NKT-like cell population, due to the presence of NK cell receptors, can exhibit properties different from conventional CD56^−^ T cells. These cells have not changed the surface expression of CD57 after the Sputnik V vaccination, but showed signs of activation according to the HLA-DR^+^ phenotype, similar to the NK cells. However, unlike NK cells, the NKT-like subset demonstrated downregulation of NKG2A expression, similar to T cells.

## 5. Conclusions

Based on the results of the study, we can conclude that both T and NK lymphocytes are implicated in response to the Sputnik V vaccine in different aspects. The progressive contraction of senescent CD57^+^ T cell fraction and, accordingly, an increase in the proportion of replicative active CD57^−^ cells, together with a decrease in HLA-DR and NKG2A expressing T cell proportions was observed after the vaccination. In turn, a short increase in frequencies of NK cells with an activating phenotype (HLA-DR, CD38, NKG2D) was pronounced in 3 days after prime-boost Sputnik V administration. Some phenotypic shifts were observed differently in SARS-CoV-2-naive and -recovered groups, which is an intriguing finding of the study. Despite the small sample size of donors, which can be considered as the main limitation of this work, in this study, we have analyzed in dynamics individual phenotypic changes in peripheral blood samples collected after the immunization. The analysis was performed in two groups of comparison: individuals with naturally acquired immunity against SARS-CoV-2 and without COVID-19 experience. The availability of “naive patients” in the background of waves and new strains of SARS-CoV-2 infection is the great value of this study. Main findings of the study are Sputnik-V-vaccination-associated with slight temporal non-specific activation of NK cell immunity and longer influence on the T cells senescence phenotype. The precise mechanisms underlying the revealed changes may be studied in further works.

Currently, we are seeing a decrease in the spread of COVID-19 in the world. However, certain groups of people with weak immunity, lung disease and others still need to have preventive vaccination. We have shown that Sputnik V, along with high immunogenicity, does not lead to significant phenotypic shifts among NK cells and T cells. New SARS-CoV-2 strains are constantly appearing. It is promising to adapt the antigen components on the adenoviral platform. It would be interesting to compare how the dynamics of the indicators analyzed in this study depend on different antigen loads.

## Figures and Tables

**Table 1:** The panels of fluorochrome-conjugated monoclonal antibodies used in the study.

Fluorochrome	Panel 1	Panel 2	Panel 3
Vio-blue/BV421	CD56	CD56	CD56
Vio-green		CD45	
FITC	NKG2C	KIR2DL2/DL3	CD38
PE	NKG2A	NKG2D	NKp30
Per-Cp/PE-Vio615	CD45	NKG2A	CD45
PE-Cy7/PE-Vio-770	HLA-DR	CD14	CD14
APC	CD3	CD16	PD-1
APC-Cy7/APC-Vio-770	CD57	CD3	CD3

**Table 2:** The levels of serum N-specific and RBD-specific SARS-CoV-2 IgGs before vaccination and post-vaccination RBD-specific IgGs 3 days and 21 days after the second Sputnik V dose administration. The results are shown as a fold-increase related to the negative optical density value measured by ELISA. A ratio greater than 1 was considered positive.

Volunteers: SARS-CoV-2-Naive (Green) SARS-CoV-2-Recovered (Red)	N-Specific IgGs before Vaccination	RBD-Specific IgGs before Vaccination	RBD-Specific IgGs 3 Days after 2nd Dose	RBD-Specific IgGs 21 Days after 2nd Dose
#3	0.1	0.24	11.2	15.3
#4	0.1	0.6	11.5	8.2
#6	0.4	0.2	2.9	8.6
#7	0.6	0.2	2.6	9.1
#9	0.2	0.22	14.1	14.4
#10	0.4	0.3	10.9	15.1
#11	0.3	0.18	6.1	11.3
#15	0.4	0.25	5.5	14.2
#16	0.4	0.32	11.9	16.6
#1	1.0	1.5	13	13.2
#2	1.1	1.7	11.2	13.3
#5	3.2	3.2	14.9	14.9
#8	0.6	2.9	15.3	17.5
#12	2.1	3.7	15.1	15.7
#13	4.6	4.1	12	15.2
#14	5.1	2.6	15.4	15.7

## Data Availability

The datasets generated and analyzed during the current study are available from the corresponding author on reasonable request.

## Supplementary Materials

The following supporting information can be downloaded at: https://www.mdpi.com/article/10.3390/vaccines11061047/s1, Figure S1: The proportion of T and NK cells before the vaccination and after the second dose of the Sputnik-V vaccine in all donors, including SARS-CoV-2-naive and recovered subgroups; Figure S2: Surface expression of CD57 molecule on NK cells before and after administration of the second dose of the Sputnik V vaccine in all the donors, and in the SARS-CoV-2-naive and recovered subgroups separately; Figure S3: Surface expression of HLA-DR on NK cells before and after administration of the second dose of the Sputnik V vaccine in all the donors, and in the SARS-CoV-2-naive and recovered subgroups separately; Figure S4: Surface expression of CD38 on T and NK cells after administration of the second dose of the Sputnik V vaccine in all the donors, including SARS-CoV-2-naive and recovered subgroups; Figure S5: Surface expression of inhibitory receptors on NK cells before and after administration of the second dose of the Sputnik V vaccine in all the donors, SARS-CoV-2-naive and recovered subgroups separately; Figure S6: Surface expression of activating receptors on conventional CD56-T cells and NKT-like subset before and after administration of the second dose of the Sputnik V vaccine in all the donors, including SARS-CoV-2-naive and recovered subgroups; Figure S7: Surface expression of NKp30 on CD56dim and CD56bright subsets of NK cells before and after administration of the second dose of the Sputnik V vaccine in all the donors, including SARS-CoV-2-naive and recovered subgroups; Figure S8: Surface expression of NKG2C+ и NKG2C+ CD57+ on NK cells before and after administration of the second dose of the Sputnik V vaccine in all the donors, including SARS-CoV-2-naive and recovered subgroups.
