# Supplementary material for: Phenotypic Changes in T and NK Cells Induced by Sputnik V Vaccination
Source: Vaccines (Basel). 2023 May 31;11(6):1047. doi: 10.3390/vaccines11061047 (PMC10303265; doi:10.3390/vaccines11061047)
Supplement: Supplementary file 1 [file vaccines-11-01047-s001.zip › vaccines-2384938-supplementary.pdf]

Supplementary file

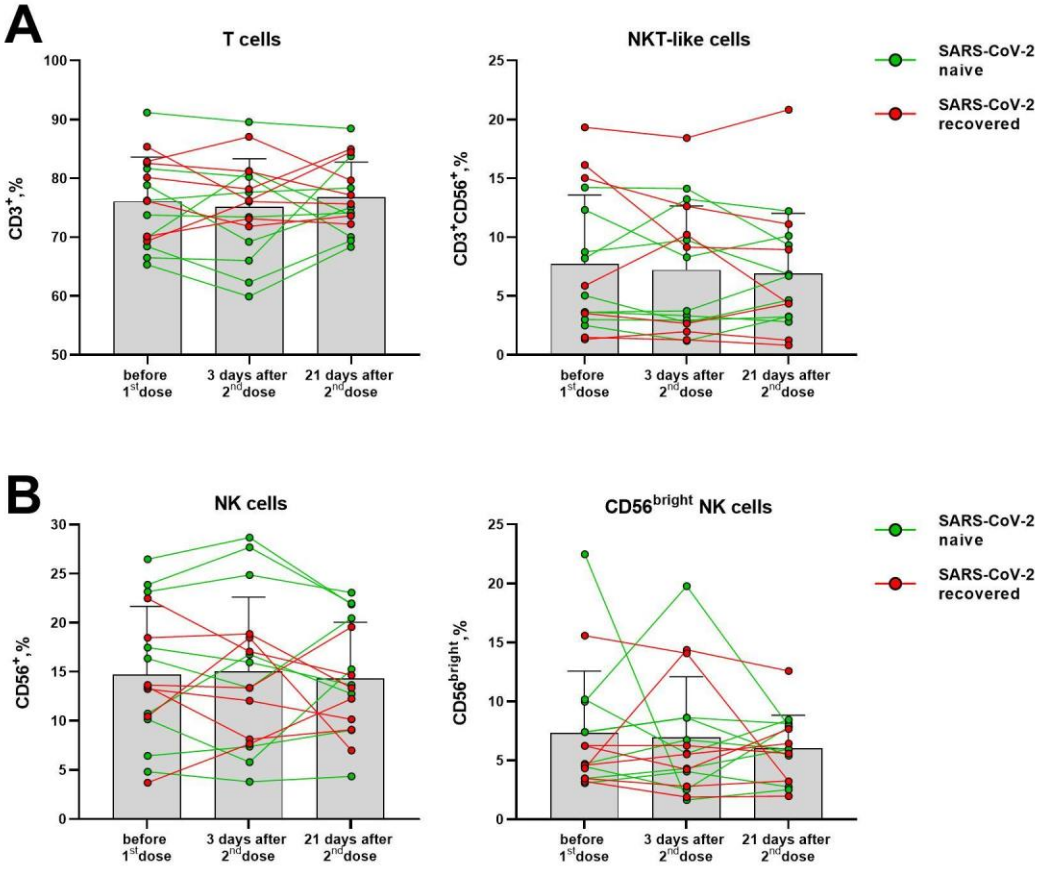

Supplementary Figure S1. The proportion of T and NK cells before the vaccination and after the second dose of the Sputnik-V vaccine in all donors, including SARS-CoV-2-naive and recovered subgroups. (A) The proportion (%) of $CD3^{+}$ T cells and NKT-like subset. (B) The proportion (%) of NK cells and $CD56^{bright}$ subset. Data are presented as mean±SD with individual values, values from the same donor are linked with color lines.

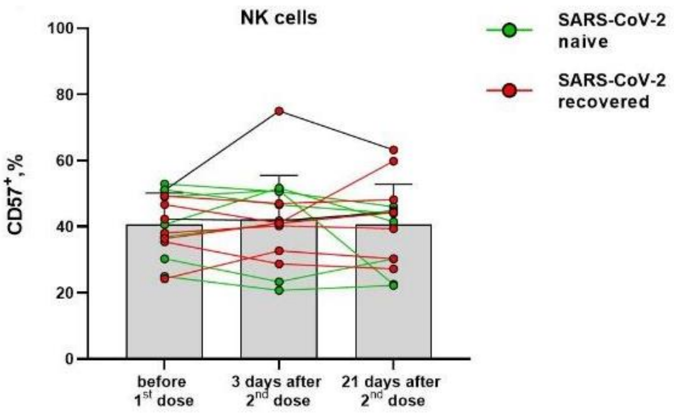

Supplementary Figure S2. Surface expression of CD57 molecule on NK cells before and after administration of the second dose of the Sputnik V vaccine in all the donors, and in the SARS-

CoV-2-naive and recovered subgroups separately. Data are presented as mean±SD with individual values, values from the same donor are linked with color lines.

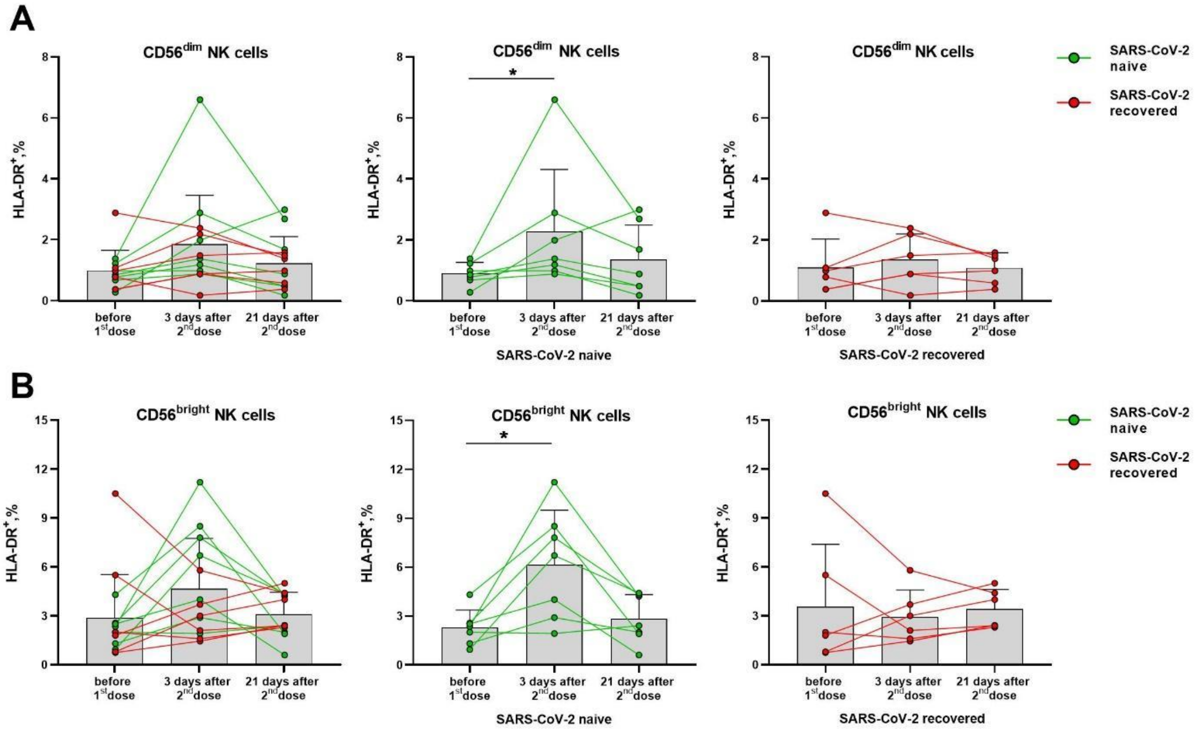

Supplementary Figure S3. Surface expression of HLA-DR on NK cells before and after administration of the second dose of the Sputnik V vaccine in all the donors, and in the SARS-CoV-2-naive and recovered subgroups separately. (A) The proportion (%) of HLA-DR^+ cells in the $CD56^{dim}$ NK cell subset. Data are presented as mean±SD with individual values, values from the same donor are linked with color lines.

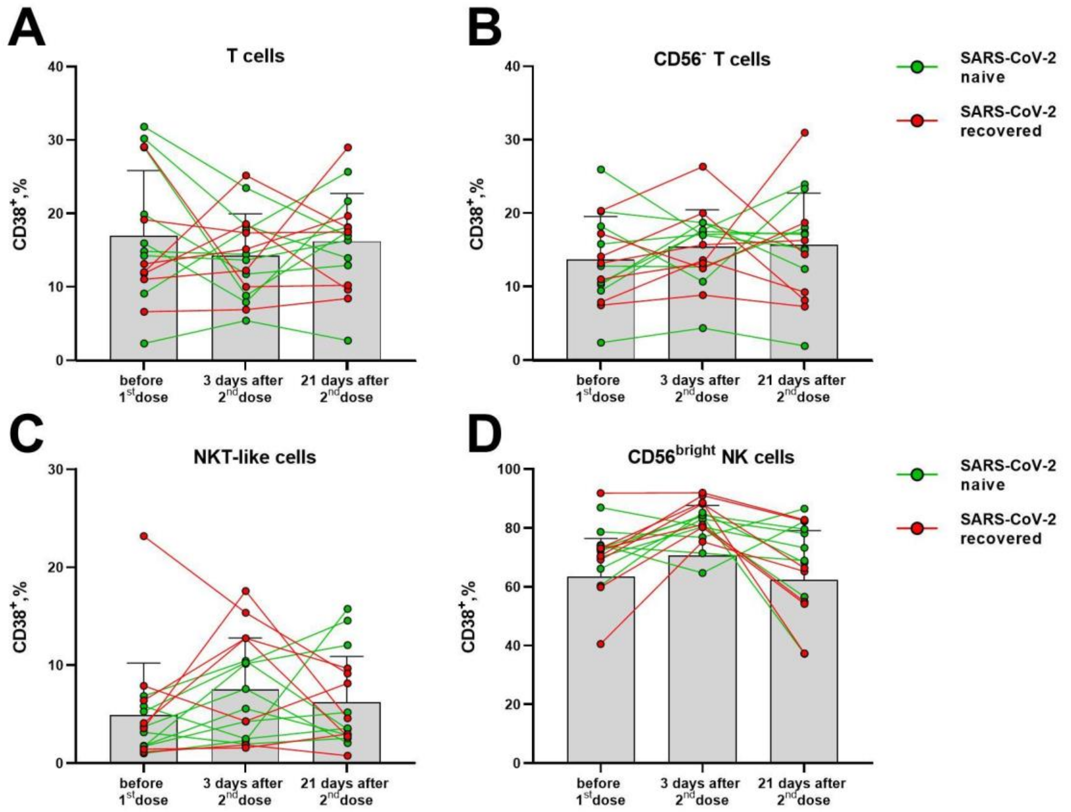

Supplementary Figure S4. Surface expression of CD38 on T and NK cells after administration of the second dose of the Sputnik V vaccine in all the donors, including SARS-CoV-2-naive and recovered subgroups. (A) The proportion (%) of $CD38^+$ cells in the $CD3^+$T cells. (B) The proportion (%) of $CD38^+$ cells in the $CD3^+CD56^-$ T cells. (C) The proportion (%) of $CD38^+$ cells in the NKT-like subset. (D) The proportion (%) of CD38- expressing CD56 ^{bright} NK cells. Data are presented as mean±SD with individual values, values from the same donor are linked with color lines.

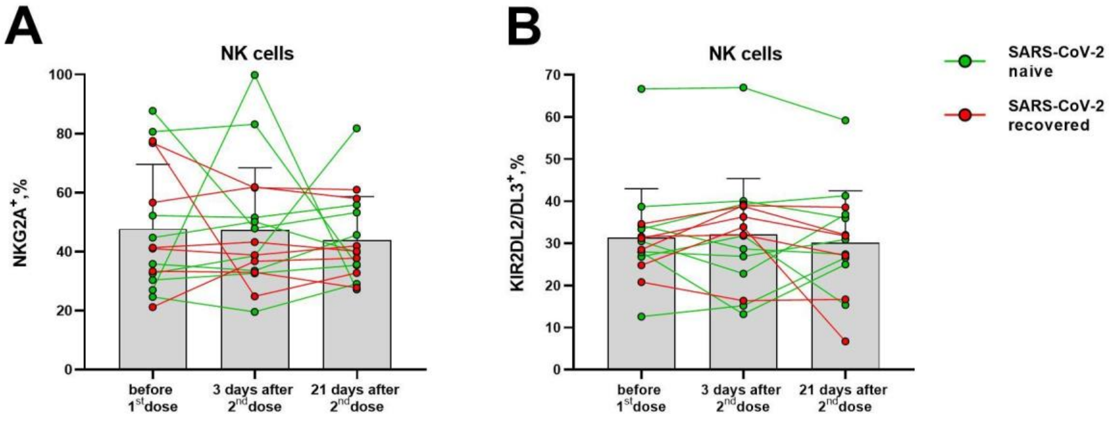

Supplementary Figure S5. Surface expression of inhibitory receptors on NK cells before and after administration of the second dose of the Sputnik V vaccine in all the donors, SARS-CoV-2-naive and recovered subgroups separately. (A) The proportion (%) of NKG2A+ cells. (B) The proportion (%) of KIR2DL2/3^+ cells. Data are presented as mean±SD with individual values, values from the same donor are linked with color lines.

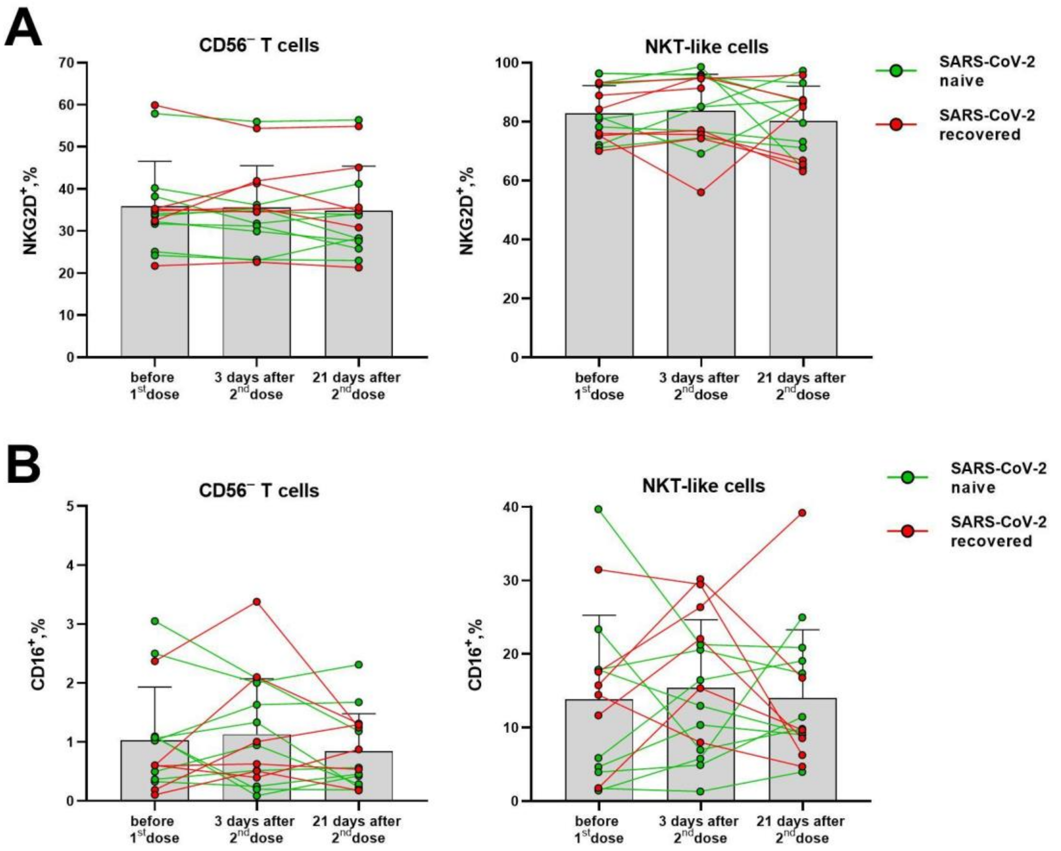

Supplementary Figure S6. Surface expression of activating receptors on conventional CD56^{-} T cells and NKT-like subset before and after administration of the second dose of the Sputnik V vaccine in all the donors, including SARS-CoV-2-naive and recovered subgroups. (A) The proportion (%) of NKG2D^{+} cells. (B) The proportion (%) of CD16^{+} cells. Data are presented as mean±SD with individual values, values from the same donor are linked with color lines.

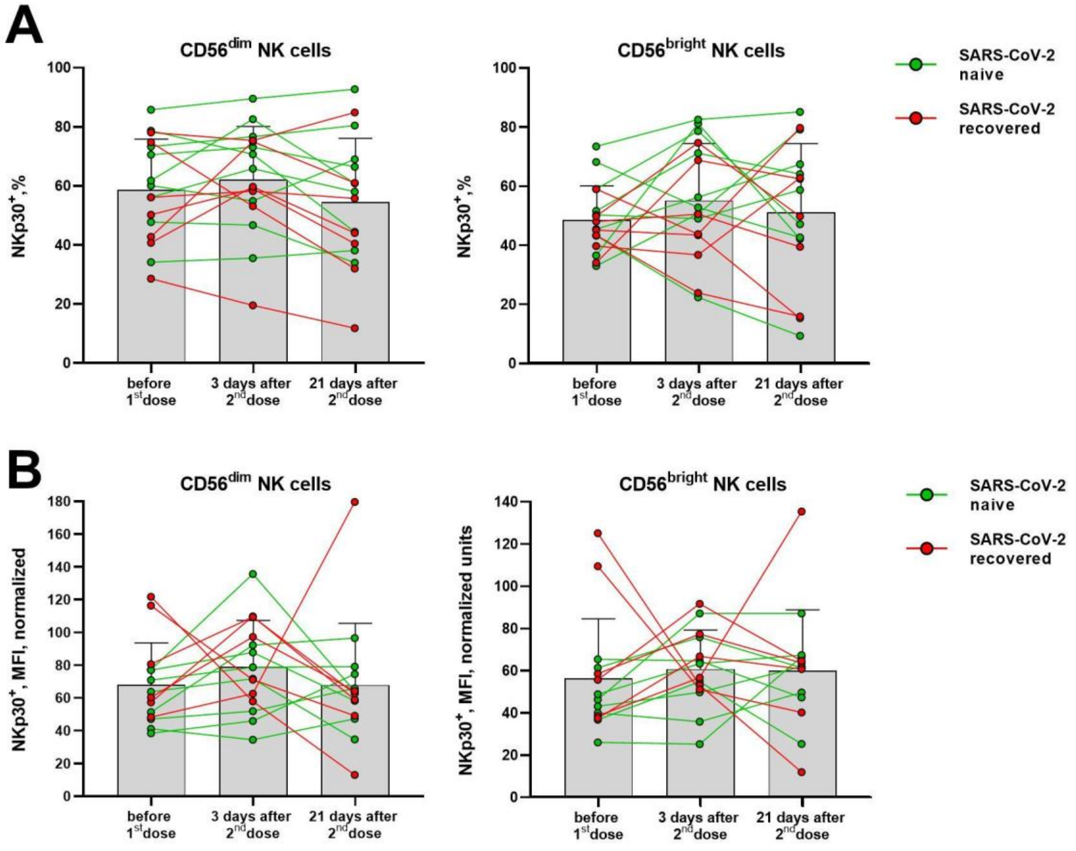

Supplementary Figure S7. Surface expression of NKp30 on CD56^{dim} and CD56^{bright} subsets of NK cells before and after administration of the second dose of the Sputnik V vaccine in all the donors, including SARS-CoV-2-naive and recovered subgroups. (A) mean of frequencies (%) of NKp30^{+} cells. (B) Normalized mean fluorescent intensity of NKp30^{+} levels. Data are presented as the mean±SD with individual values, values from the same donor are linked with color lines.

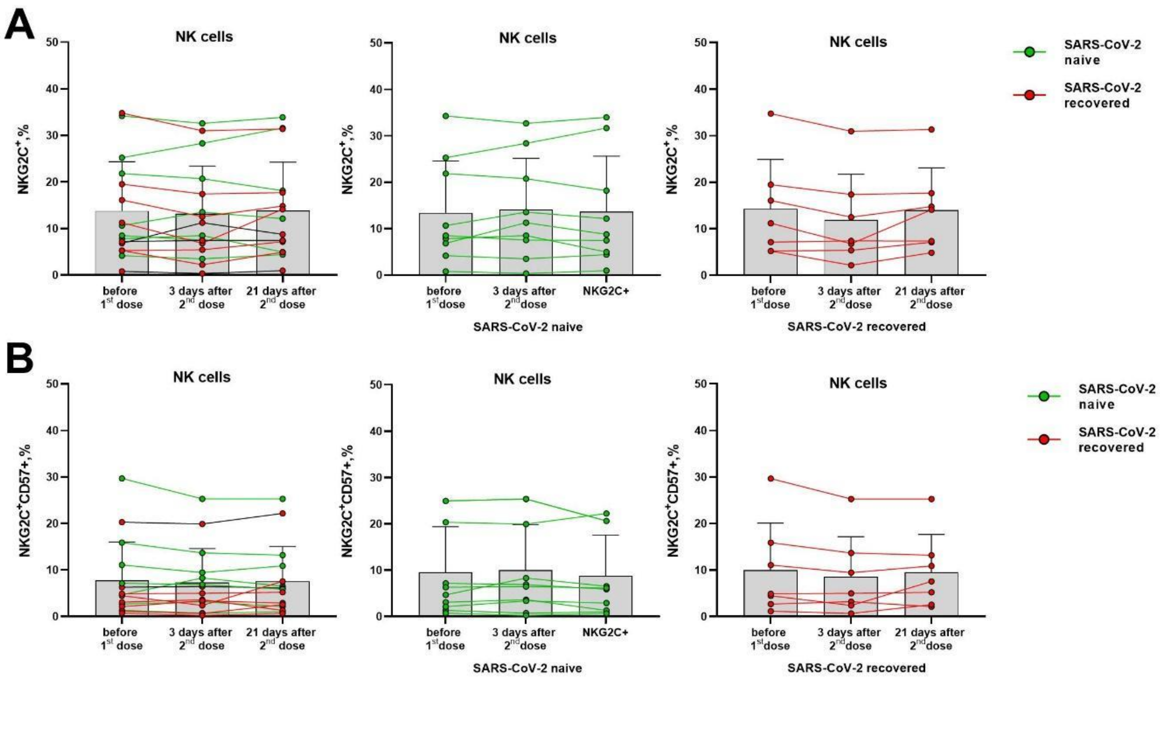

Supplementary Figure S8.Surface expression of NKG2C^{+} and NKG2C^{+} CD57^{+} on NK cells before and after administration of the second dose of the Sputnik V vaccine in all the donors, including SARS-CoV-2-naive and recovered subgroups. (A) Mean of frequencies (%) of NKG2C^{+} NK cells; (B) NKG2C^{+}CD57^{+} NK cells. Data are presented as the mean±SD with individual values, values from the same donor are linked with color lines.
